# A Rationale for the Use of F18-FDG PET/CT in Fever and Inflammation of Unknown Origin

**DOI:** 10.1155/2012/165080

**Published:** 2012-12-17

**Authors:** H. Balink, H. J. Verberne, R. J. Bennink, B. L. F. van Eck-Smit

**Affiliations:** ^1^Department of Nuclear Medicine, Medical Center Leeuwarden, 8901 BR Leeuwarden, The Netherlands; ^2^Department of Nuclear Medicine, Academic Medical Center, University of Amsterdam, The Netherlands

## Abstract

This review focuses on the diagnostic value of hybrid F18-FDG Positron Emission Tomography/Computerized tomography (PET/CT) in fever of unknown origin (FUO) and inflammation of unknown origin (IUO). Due to the wide range of possible causes both FUO and IUO remain a clinical challenge for both patients and physicians. In addition, the aetiology of IUO shows the same variation in diseases as the FUO spectrum and probably requires the same diagnostic approach as FUO. There are numerous historically used diagnostic approaches incorporating invasive and non-invasive, and imaging techniques, all with relative high specificity but limited sensitivity. This hampers the generalization of these diagnostic approaches. However, recently published reports show that F18-FDG PET/CT in FUO and IUO has a high sensitivity and a relative non-specificity for malignancy, infection and inflammation. This makes F18-FDG PET/CT an ideal diagnostic tool to start the diagnostic process and to guide subsequent focused diagnostic approaches with higher specificity. In addition, F18-FDG PET/CT has a relative high negative predictive value. Therefore F18 FDG PET/CT should be incorporated in the routine diagnostic work-up of patients with FUO and IUO, preferably at an early stage in the diagnostic process.

## 1. Introduction

### 1.1. Definition of Fever of Unknown Origin

Fever of unknown origin (FUO) was first used by Kiefer and Leard in their book “prolonged and perplexing fevers”. In their seminal article from 1961 Petersdorf and Beeson defined (FUO) as: (1) an illness of at least 3 weeks' duration, (2) with fever; body temperature higher than 38.3°C (101°F) on several occasions, and (3) no established diagnosis after 1 week of hospital investigation [1]. A period of 3 weeks was chosen to eliminate self-limited viral illnesses and to allow sufficient time to complete the appropriate diagnostic procedures.

Since 1961 health care has shifted from inpatient oriented healthcare to a more outpatient setting, and in response to the increasing sophistication of medical technology, Durack and Street proposed a change towards the required duration of investigation before qualifying a fever as FUO; at least 3 days in hospital or at least three outpatient visits [2]. 

### 1.2. Aetiology of FUO

Although the definition of FUO suggests that the fevers remain of unknown origin, most of the FUOs have a pathophysiological basis. Based on these pathophysiological disorders, the spectrum of FUOs may be divided into four general categories: (1) infections, (2) malignancies, (3) noninfectious inflammatory diseases, or (4) miscellaneous disorders including drug-related fever, habitual, hyperthermia, and factitious fever. The phrase “noninfectious inflammatory diseases” is semantic for rheumatic diseases, autoimmune diseases, systemic diseases, collagen vascular diseases or vasculitides or vasculitis, connective tissue diseases, and granulomatous diseases. Over the past 40–50 years the proportion of cases of FUO caused by infections and neoplasms has decreased. This is most likely due to the relatively easy detection of solid tumors and abnormal lymphnodes via dramatically improved diagnostic properties of ultrasonography and computed tomography (CT) [3]. 

Durack and Street proposed a further diversification: classical FUO, versus nosocomial FUO, neutropenic FUO, and HIV-associated FUO. Opposite to classical FUO, in the latter three groups infections represent the most common category. 

As there are more than 200 different reported causes for FUO, the differential diagnosis is the most extensive in medicine [4]. A systemic review from 2003 reported that approximately 1.5–3% of all hospitalized patients deal with FUO, and in addition 12–35% of these patients die of FUO-related problems [3].

### 1.3. Diagnosis of FUO

FUO is frustrating for both patient and physician because the diagnostic work involves several noninvasive and invasive procedures and may fail to reach a diagnosis in up to 50% of cases [5–7]. Not only early detection of infections but also early diagnosis or exclusion of malignancy is of utmost importance for optimal patient management. The number of patients with FUO who are eventually diagnosed with malignant disease are nowadays rather small (usually less than 10% of cases in modern FUO populations in Western countries). Therefore a good accuracy for diagnosing malignant disease should also be an important requirement of any diagnostic approach (i.e., imaging technique) in these patients.

### 1.4. Diagnostic Strategy for FUO

In the vast literature that exists on FUO there is no diagnostic gold standard against which other diagnostic tests may be measured. For this reason there is disagreement in the published diagnostic algorithms and as to which investigations should constitute a comprehensive diagnostic workup [3, 8, 9]. 

In lack of a standardized diagnostic strategy, there is also disagreement whether or not the diagnostic process should be guided by the so called “potential diagnostic clues” (PDC). These PDC emerge from medical history, physical examination, and baseline tests. PDC are defined as all localizing signs, symptoms, and abnormalities potentially pointing towards a diagnosis. In a review of the literature from 1995 to 2004, the diagnoses obtained in patients with PDC were significantly higher than in patients without PDC (72% versus 30%) [9]. In a prospective multi-centre study however, 15 PDC, on average, were identified per patient, of which 81% proved to be misleading. The remaining 19% of PDC contributed to the final diagnosis, but PDC alone did not directly lead to a diagnosis in any of the patients [10]. 

### 1.5. Inflammation of Unknown Origin (IUO)

Remarkably little literature covers unexplained inflammatory syndromes without persisting fever (i.e., inflammation of unknown origin (IUO)). Perrin et al. described a retrospective series of 47 patients (median age, 67), gathered over a 7 years period, with an inflammatory syndrome that remained without diagnosis during hospital admission (for a median of 20 days). The threshold for C-reactive protein (CRP) values was 15 mg/L. During followup, an ultimate diagnosis was established in 14 patients (30%), most frequently polymyalgia rheumatica or giant cell arteritis [11]. A more recent study in 73 patients reported that low-grade fever (body temperature between 37.5 and 38.3°C) required the same diagnostic approach as FUO because there was no relationship between body temperature values and the severity of the underlying diseases, and the aetiological spectrum was also in line with the causes related to FUO [12]. 

**Figure 1:**
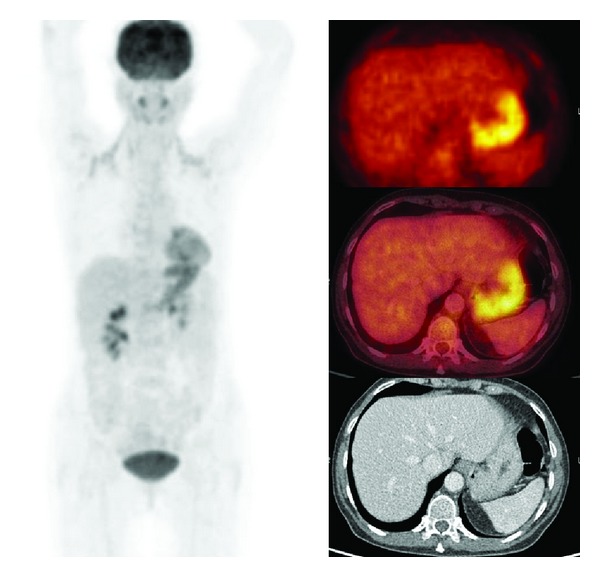
Fifty-nine years old female male with anaemia and weight loss (CRP 56 mg/L, ESR 42 mmh/h) and vague pain in the area of the lower thoracic vertebral column. Chest radiography and US abdomen showed no abnormalities. CT of thorax and abdomen showed no abnormalities either. The F18-FDG PET/CT images showed high uptake in the gastric wall. Gastroscopy revealed gastritis with superficial ulcerae. Cultures of biopsy specimen were positive for *Helicobacter pylori*. After treatment with amoxicillin, clarithromycin, pantoprazole, and ferrous fumarate both CRP and ESR normalized and hemoglobin values increased to normal values. Note increased uptake bilateral in the neck due to brown adipose tissue.

**Figure 2:**
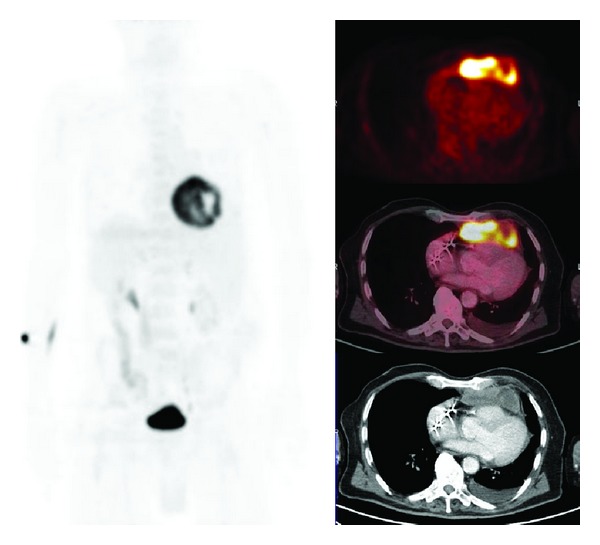
Seventy-seven years old male—with a pacemaker since a year—presented with fatigue, coughing, 5 kg weight loss, and short episodes of sub-febrile temperatures. Initial diagnostic workup showed anaemia, and increased inflammatory markers (CRP 93 mg/L, ESR 60 mm/h); blood cultivations were negative. Chest radiography showed no abnormalities. In further search for an explanation an F18-FDG PET/CT was performed. The PET/CT showed increased uptake of F18-FDG in a large precardiac mass. Biopsy of the large precardiac mass showed a diffuse large cell B-cell lymphoma (DLBCL). According to the guidelines and age-related, patient got only palliative treatment, starting with steroids.

**Figure 3:**
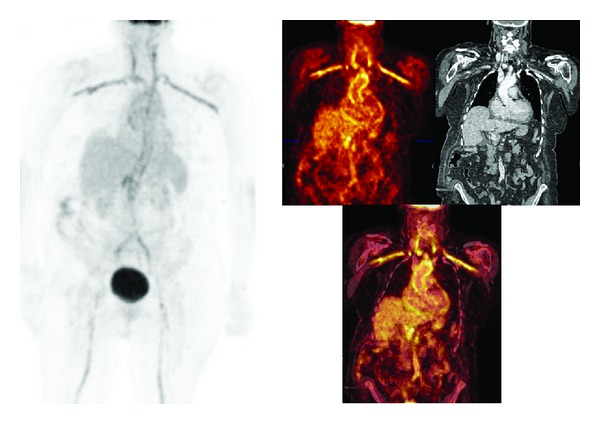
Seventy-three year old woman with belching complaints and recurrent pain between the shoulder blades with increasing intensity. Coronary artery disease as a probable cause was excluded at the cardiology department. Laboratory revealed a CRP of 224 mg/L with an ESR of 64 mm/h. Chest radiography showed an increased cor/thorax ratio. US abdomen no abnormalities. CT of thorax and abdomen showed signs of left pleural effusion and pericardial fluid, which was considered not enough for biopsy by the cardiologist. Virus serology was negative. Histopathology of pleural fluid showed signs of inflammation, and no malignancy. Histopathology of duodenal biopsy specimen without abnormalities. F18-FDG PET/CT showed pathologic uptake in the wall of the aorta and its main branches, both thoracic and abdominal. Patient was diagnosed as having large vessel vasculitis and accordingly treated with prednisolone and became free of complaints, CRP and ESR normalized.

Vanderschueren et al. prospectively collected a series of 57 consecutive patients with IUO, defined as: (1) an illness of at least 3 weeks' duration, (2) with signs of inflammation but with temperatures below 38.3°C, CRP > 30 mg/L and/or ESR > age/2 in ♂ or (age + 10)/2 in ♀ on >3 occasions), and (3) uncertain diagnosis despite appropriate investigations after at least 3 days of hospital investigation or 3 outpatient visits. These patients defined with IUO were compared with a contemporary set of age- and sex-matched patients with classical FUO, according to the definition of Durack and Street. The authors found that the diagnostic yield, case-mix, contribution of F18-FDG PET, and vital outcome were similar in both groups [13]. Therefore it has been suggested to delete the 38.3°C boundary from the original criteria by Petersdorf and Beeson [5, 14]. In short, the aetiology of IUO shows the same variation in diseases as the FUO spectrum and probably requires the same diagnostic approach as FUO. In Figures 1, 2, and 3 examples of the variety in aetiology are shown. 

## 2. Diagnosis

Although the diagnostic approach in FUO has not been uniform it has always included a thorough medical history, careful physical examination, laboratory tests, and a mix of imaging techniques. A thorough and repeated medical history is important, including information about alcohol intake, medications, occupational exposures, pets, travel, familial disorders, and previous illnesses [15–17]. 

The specific findings of a thorough physical examination that have led to a diagnosis in FUO are numerous and diverse. Examples included slight enlargement of the thyroid (thyroiditis), periodontal disease or loose teeth (dental abscess), thickened temporal artery (temporal arteritis), cardiac murmur that changes with position (atrial myxoma), and widespread hyperpigmentation (Whipple's disease). But for instance lymphadenopathy either in children or elderly has not correlated with specific illnesses or a positive biopsy [18, 19]. Although the findings of a thorough physical examination are often misleading, they may help in limiting the list of probable diagnoses (high specificity).

Use of clinical fever curves were reported to be useful as specific fever patterns have been ascribed to many of the causes of FUO [20]. Unfortunately, in most case series, the height, pattern, or duration of fever did not relate to diagnosis [6]. 

In the absence of a consensus on the best/optimal diagnostic strategy for FUO, patients undergo a plethora of diagnostic tests ranging from relatively noninvasive to an exploratory laparotomy. The following sections describe the variety of diagnostic tests (noninvasive, invasive, and imaging modalities) described in the literature used to establish the aetiology of FUO.

### 2.1. Noninvasive Procedures

#### 2.1.1. Fundoscopy

Retinal abnormalities associated with infections include Roth's spots (white-centred haemorrhages) with infective endocarditis, yellowish-white choroidal lesions with tuberculosis and certain disseminated fungal infections, and active retinitis caused by disseminated toxoplasmosis or cytomegalovirus in immunocompromised patients. Patients with malignancies may have choroidal metastases, usually from a breast or lung malignancies. Leukaemia can cause intraretinal haemorrhages, Roth's spots, and leukemic infiltrates. Various forms of vasculitis produce cotton-wool exudates, intraretinal haemorrhages, and vascular occlusive disease, while sarcoidosis can cause perivascular sheathing (“candle-wax drippings”) and choroidal nodules [7]. Of importance is to realize that each of these diseases may exist without abnormalities at fundoscopy. 

#### 2.1.2. Leg Doppler Imaging

Three patient series reported a deep vein thrombosis as the cause of FUO in 2% to 6% of patients, despite the small percentages leg Doppler imaging is safe and may identify a treatable cause [21–23]. 

### 2.2. Invasive Diagnostic Procedures

Most FUO patients underwent at least one of the following procedures, if the source of the fever remained elusive after a scala of all possible noninvasive diagnostic procedures, even though the yield was known to be moderate.

#### 2.2.1. Bone Marrow Aspiration

This has been proven of little value as a screening procedure, in the absence of PDC for a bone marrow disorder. In addition bone marrow aspiration has not proven to be useful in patients with FUO and anemia [24]. 

#### 2.2.2. Percutaneous Liver Biopsy

An early or “blind” liver biopsy can be helpful for the diagnosis of granulomatous hepatitis [25]. However, as granulomatous hepatitis represents an aspecific histological reaction to infections, neoplasms or drugs, it is considered a group of diseases. Therefore, it cannot be put forward as a final diagnosis [5]. Only in HIV-infected patients with FUO in combination with hepatosplenomegaly and increased alkaline phosphatase levels, an early liver biopsy has been described as a useful diagnostic technique [26]. 

#### 2.2.3. Skin and Muscle Biopsy

The diagnostic yield of these biopsies has only been proven when performed in patients with skin abnormalities and/or abnormal electromyography [27–29]. 

#### 2.2.4. Temporal Artery Biopsy

The only biopsy that may be rewarding in the absence of prior localising information is temporal artery biopsy in elderly patients with a high CRP/ESR, although it can be false negative in 15–70% of the cases, which may delay the diagnosis [30]. Temporal arteritis due to giant cell arteritis (GCA) is an important cause of FUO in patients older than 50 years. In one patient series the contribution of GCA temporal arteritis was as high as 15% of the cases [31]. In recent series, patients with increased F18-FDG uptake in the thoracic aorta and its main branches, suggestive for GCA, also underwent temporal artery in 39% of these cases, only half of these patients had a positive temporal artery biopsy [32]. 

#### 2.2.5. Bronchoalveolar Lavage (BAL)

Mostly performed in patients with an abnormal chest radiography; a myriad of pulmonary problems is associated with abnormal imaging findings (mass, cavitary lesion, infiltrates, etc.). In comparison to BAL lung fine-needle aspirate (LFNA) is the superior method for the cytologic diagnosis of pulmonary pathology [33]. BAL was only reported useful in advanced HIV infection with alveolar lymphocytosis [34]. 

#### 2.2.6. Exploratory Laparotomy

In the absence of localising features, exploratory laparotomy is considered obsolete these days. 

In general, the above mentioned (non-)invasive investigations have a high specificity, but are hampered by their relatively low negative predictive value. Therefore none of these investigations can reliably exclude malignancy, focal inflammatory disease, or infection. This limitation is essential and may be overcome with the use of imaging techniques; especially F18-FDG PET/CT seems to be very promising.

### 2.3. Imaging Techniques

Imaging techniques include anatomical imaging modalities like radiographs, ultrasonography (US), computerized tomography (CT), magnetic resonance imaging (MRI). Conventional nuclear medicine scintigraphy includes planar/total body scintigraphy and single photon computerized photography (SPECT) and positron emission tomography (PET). All these techniques have specific advantages and disadvantages. In order to understand the evolving role of hybrid F18-FDG PET/CT in relation to the other imaging techniques, it is important to be aware of their respective advantages and disadvantages.

#### 2.3.1. Plain-Film Radiography

Chest radiography is not only valuable for disclosing intrathoracic disorders but also for suggesting intra-abdominal pathology. In most patients with subphrenic, splenic, hepatic, and pancreatic abscesses, an ipsilateral finding of atelectasis, an elevated hemi-diaphragm, or a pleural effusion is present, and in a few cases an intra-abdominal mass is visible on the sub-diaphragmatic part of the film. Plain-film radiography can show typical findings of soft tissue swelling, although they may not be apparent during the early phases of disease. It usually takes 2 to 3 weeks for an osseous lesion to become visible on plain-film radiography because significant loss of bone density must occur before such changes become apparent [35]. 

#### 2.3.2. Ultrasonography (US)

US is widely available, quick, inexpensive, and not associated with radiation exposure. The spatial resolution may reach below 1 mm and US can be used to obtain functional information to a limited extent (e.g., blood flow by Doppler ultrasonography). The disadvantage may be that the results are highly operator-dependent. The penetration and reflection of the sound waves in tissue may be hindered by gas (bowel) or dense structures (bone), and structures deep within the body may be difficult to visualize because the image quality suffers from the longer wavelengths used for deep imaging. In adults, the failure of ultrasound to detect many liver, spleen, and intra-peritoneal abscesses precludes reliance on this examination.

#### 2.3.3. US of the Heart (Echocardiography)

Echocardiography can help to diagnose infective endocarditis in demonstrating vegetations. The sensitivity of the trans-thoracic approach is approximately 55–68%, and of the trans-esophageal approach approximately 90–94% [36]. Besides infective endocarditis US can detect other causes of FUO related to the heart such as myxoma, sarcoidosis, or other infiltrative diseases.

#### 2.3.4. Computerized Tomography (CT)

CT is highly reproducible, has an excellent spatial resolution, and, although more expensive than ultrasonography, is still relatively inexpensive. The examination time is short, generally less than 5–10 min. A disadvantage is exposure of the patient to radiation, the substantial radiation to the organs examined limits its use at frequent intervals. Furthermore, the use of contrast medium to enhance image contrast may be limited or impossible in patients with impaired renal function or previous allergic reactions. Also CT has a lack of functional information. False-negative CT results have occasionally been reported, even with abscesses in solid organs, due to distortions of normal anatomy, small abscess size, or failure to use both oral and intravenous contrast agents [37]. In neutropenic patients with FUO low-dose multislice CT is useful for the early detection of pneumonia—at relatively low cost and radiation burden [38]. 

#### 2.3.5. Magnetic Resonance Imaging (MRI)

MRI is also characterized by a high spatial resolution; it provides excellent structural resolution for visualizing advanced stages of disease. It has some potential to obtain functional information, and causes no radiation exposure. It has also become widely available but is prone to movement artifacts because of the relatively long examination time. Furthermore, there are limitations to the scanning of patients with pacemakers, implants, and other devices, and the procedure is relatively expensive. In general MRI is (compared to CT) more useful for the evaluation of internal structures such as the bone marrow, muscles, tendons, ligaments, cartilage, and small organs such as the prostate gland, testes, cervix, and uterus [39]. 

In general radiological techniques, including CT, MRI, and ultrasonography show anatomical changes and consequently, malignant, infectious, and inflammatory foci cannot be detected in an early phase because of the lack of substantial anatomical changes at this time. After surgery or other therapeutic interventions like radiotherapy, discrimination of active malignant, infectious or inflammatory lesions from residual anatomical changes is often difficult.

Therefore, functional and metabolic imaging with scintigraphic methods plays an important complementary role in the diagnostic process of patients with FUO. The radiation exposure is low but, dependent on the radiotracer used, may reach the radiation exposure of an abdominal CT scan (2–6 mSv). Conventional planar imaging had the disadvantage that the exact localization of affected sites in most anatomic regions of the body was limited, the introduction of hybrid SPECT/CT has overcome this disadvantage [40]. 

A wide variety of conventional radiopharmaceuticals/radiotracers has been tested, but currently only a few radiopharmaceuticals are in general use and/or commercially available for imaging of malignancy, infection and inflammation. These include autologous white blood cells (WBCs (leukocytes)) labelled with Tc99m or In111, Tc99m-labeled bisphosphonates such as methylene diphosphonate or hydroxymethylene diphosphonate, Ga67-citrate, Tc99m antigranulocyte antibodies, Tc99m-labelled nanocolloids, and Tc99m- or In111-labeled proteins, such as human polyclonal immunoglobulin (HIG) or albumin [41]. Recently introduced radiolabelled antibodies for the detection of sites of infection were withdrawn from the market because of serious side effects in patients [42]. 

The main disadvantage of conventional radiopharmaceuticals is that each (apart from Ga67), covers only a part of the spectrum of possible diagnoses in the broad setting of FUO and IUO. There are also disadvantages like handling of potentially infected blood products (WBC), high radiation burden, and poor imaging characteristics (WBC, Ga67) and the long-time span, 2-3 days, between injection and diagnosis (Ga67).

Ga67-citrate scintigraphy was until recently the most used radiopharmaceutical for imaging in patients with FUO/IUO and was considered the “gold standard,” because of its ability to detect both acute and chronic infectious and inflammatory conditions and some neoplasms [43, 44]. The clinical application is limited though, the specificity is decreased due to hepatobiliary excretion and the accordingly rather high physiological bowel activity and excretion. Also physiological uptake in active cortical bone remodelling hampers the accuracy [45, 46]. In 145 cases of FUO a final diagnosis with Ga67 was established in 99 (68%), only 42 of the abnormal scans (29% of the total number of scans) were considered helpful in diagnosis; therefore 49% of the abnormal scans were considered as not helpful to the diagnosis [44]. Besides, optimal imaging requires delayed imaging up to 72 hours after the injection. The unfavourable imaging characteristics, the long physical half-life (78 hours), and the high-energy gamma radiation (93–889 keV), causes a high radiation burden to the patient, contributing to the current preference for F18-FDG PET/CT.

As for ex vivo-labelled autologous leukocytes, the usefulness in many acute and several chronic infections and inflammatory diseases is widely established. However, the use of leukocyte scintigraphy in patients with FUO is scarcely reported. From a series of 166 white blood cell studies performed in clinical suspicion for sepsis, 28 cases were of unknown origin. Only 11% of studies revealed a pyogenic cause for FUO [47]. Another rather small study reported that In111-leukocyte scintigraphy was helpful in 22% of 32 patients with FUO, and with false-positive results in 4 patients (13%) [48]. In a more recent retrospective study including 31 patients with FUO In111-leukocyte scintigraphy was helpful in 19% of all cases while the probability of reaching a diagnosis was relatively high: 71% [49].

In a retrospective study of 220 In111-granulocyte scintigrams from 208 patients, 25 patients had malignant neoplasms. Among these, pathological uptake of In111 activity in malignancy was observed in only ten patients (intense activity in two patients with non-Hodgkin's lymphoma and colonic carcinoma, resp.; moderate uptake in a patient with non-Hodgkin's lymphoma, and in a patient with an ovarian carcinoma; weak activity in three patients with cerebral neoplasms; and activity within otherwise “cold” metastatic lesions of the liver in three patients) [50]. 

In a study of 117 patients with known various malignancies In111-leukocyte scintigraphy was performed in order to diagnose localized infectious disease. The accuracy for infection as comorbidity of malignancy was 91%. However, no uptake was observed in primary or secondary tumors, with the exception of accumulation of labelled leukocytes at the site of an osteolytic metastasis in one case [51]. 

### 2.4. F18-FDG Positron Emission Tomography (PET)/Computerized Tomography (CT)

The most widely used PET tracer is the glucose analogue 2-deoxy-2-(F-18) fluoro-D-glucose (F18-FDG). PET imaging in the oncology setting is based on the increased glycolytic rate in malignant cells, and overexpression of glucose transporters (GLUT-1 and -3). Intracellularly FDG is phosphorylated to FDG-6-phosphate by hexokinase. Because FDG-6-phosphate is not a suitable substrate for the glycolytic enzymes that follow, FDG-6-phosphate continues to accumulate intracellularly. Similarly many infective and inflammatory conditions can be imaged with PET due to the increased accumulation of F18-FDG by inflammatory cells and granulation tissue, as these cells use glucose as an energy source only after activation during the metabolic burst [52]. Increased FDG uptake is present in all activated leukocytes (granulocytes, monocytes as well as lymphocytes) enabling imaging of acute and chronic inflammatory processes. 

The development of hybrid PET/CT was a milestone in medical imaging. Hybrid PET/CT augments the accuracy in the workup of FUO and IUO as a result of the synergy of the anatomic-metabolic information. Hybrid PET/CT allows the use of imaging with high spatial resolution to localize and characterize tissues with increased metabolism, providing in this way a significant contribution in the process of finding the causes of pathological processes.

In the recognition that F18-FDG shows increased uptake in not only malignant cells, but also in cells involved in infectious and inflammatory processes, the possible advantages of PET and later hybrid PET/CT over other diagnostic procedures in identifying FUO aetiology were already understood several years ago. In less than 15 years its use in this field has been extensively evaluated (Tables 1 and 2). 

In 2004, Meller et al. reported superiority of F18-FDG PET compared to Ga67-citrate scintigraphy [53]. PET was helpful in 55% of patients, showing a high positive predictive value (PPV); furthermore, the authors reported on the good prognostic value of a negative PET result. Blockmans et al. also compared F18-FDG and Ga67-citrate scintigraphy in 58 patients with FUO [54]. PET was used as a second line investigation when the aetiology was not identified after medical history and clinical examination, routine laboratory tests, chest radiography, and abdominal US. PET helped to establish the final diagnosis in 24 patients, with a diagnostic yield of 41%. Lorenzen et al. studied 16 patients with FUO and elevated CRP and ESR [55]. F18-FDG was helpful in 69% of the cases. Importantly none of the other diagnostic techniques used in the PET-negative cases were able to detect the underlying cause of the fever.

In a prospective study of 19 patients with classical FUO, Kjaer et al. compared 111In-granulocyte scintigraphy and F18-FDG PET [49]. In contrast with previous results, PET was found to be useful in only 16% of cases, with a superior specificity of 111In-granulocyte scintigraphy. However, patients were only recruited from the department of infectious diseases. Therefore, as already pointed out by Bleeker-Rovers, this discrepancy with other studies may be, at least in part, attributed to selection bias: patients with classical FUO without signs of infection could be admitted to other departments that do not routinely refer patients for (111In-granulocyte) scintigraphy [66]. In 4 of 19 patients in whom no cause of the fever could be found, abnormal findings on F18-FDG PET were considered to be false positive. No additional tests were performed to exclude or confirm an underlying disease as an explanation for the found abnormalities on PET. This influences importantly the sensitivity of both techniques and particularly the specificity of F18-FDG PET.

Buysschaert et al. reported in a retrospective study of 74 patients with FUO that PET helped to establish the final diagnosis in 26% of cases; 19 scans were helpful in 39 patients with a final diagnosis [57]. In a prospective multicentre study, Bleeker-Rovers et al. evaluated the role of PET in 70 patients with FUO [8]. A diagnosis was reached in 50% of cases. PET was helpful to establish the final cause of FUO in 33% of the 70 patients, with a PPV of 70%. Furthermore, PET was more useful in patients with continuous fever compared to those with intermittent fever and was found useful in 39% of cases with elevated CRP and ESR but not in patients with normal CRP and ESR values.

In a study in 118 patients with FUO Jaruskova and Belohlavek reported that PET or PET/CT was helpful for diagnoses in 36% of the 118 patients with prolonged febrile status (76% FUO, 24% suspected postsurgical infections) [58]. (Overview of stand-alone PET results in Table 1).

The use of hybrid PET/CT adds important advantages to the use of stand-alone F18-FDG PET. As PET imaging is able to reveal functional alterations that precede the morphological changes, the integration of anatomical and morphological images allows improved interpretation of both abnormal F18-FDG uptake and suspicious morphological findings. The overall helpful contribution in the diagnostic approach with F18-FDG PET/CT increased considerably from 39% to 57% compared to stand-alone F18-FDG PET. PET scans were considered as “helpful contribution” when the PET study demonstrated a focal localized disease process, confirmed by other investigations, as being the cause of FUO. 

Thereby the overall percentage of finding a final diagnosis increased from 67% to 73% (Table 2).

It should be pointed out that most studies also stress the high negative predictive value of the hybrid technique in the assessment of FUO. A negative F18-FDG PET/CT performed after the initial workup, with the exclusion of non-focal systemic diseases, was highly indicative for establishing a wait-and-see strategy and obviated the need for further investigations.

### 2.5. Helpful Contribution of F18-FDG PET/CT in Inflammation of Unknown Origin (IUO)

The conclusion of Vanderschueren et al. in 2009 that the 38.3°C boundary of classic FUO seems arbitrary and the diagnostic approaches used in unexplained prolonged febrile disorders can be applied to unexplained prolonged inflammatory disorders was confirmed in 2010 in a paediatric setting [13, 67]. 

In children the diagnostic workup of FUO and unexplained signs of inflammation may be traumatic, including biopsies and bone marrow examinations. Without a diagnosis, there is frequently a need for a treatment with either antibiotic or steroid therapy. In a retrospective study, 47 F18-FDG PET and 30 PET/CT scans from 69 children were analysed. Children suffered from either FUO (*n* = 44) or IUO (*n* = 33). A diagnosis could be established in 32 patients (54%). Of all scans, 63 (82%) were abnormal, and of the total number of 77 PET and PET/CT scans 35 (45%) were clinically helpful. In patients with a final diagnosis, scans were found to have contributed to the diagnosis in 73%. Of the hybrid PET/CT scans, 53% were considered helpful, whereas stand-alone PET was helpful in only 40%. Laboratory, demographic, or clinical parameters of the children did not predict the usefulness of FDG PET scans. 

These results prompted to a multi-centre retrospective study that included 304 patients with clinical suspicion for large vessel vasculitis (LVV) and not fulfilling the temperature criteria for classic FUO. The suspicion for LVV was based on increased laboratory inflammation parameters (CRP, ESR) and a variety of several non-specific symptoms, such as fatigue, night sweats, weight loss, malaise, fatigue, and myalgia. Patients without typical signs of temporal arteritis (e.g., headache, jaw claudication, and scalp tenderness) were also included. PET images were considered positive for LVV in case of homogeneous smooth linear and long segmental uptake, with higher intensity compared to the liver and in the thoracic aorta and its main branches, which is considered to be a characteristic pattern of giant cell arteritis (GCA) [68]. Sixty-two (20%) were positive for LVV and 242 (80%) were negative for LVV. Interestingly, in the group with a negative result for LLV the diagnostic yield, contribution of F18-FDG PET/CT scintigraphy and case-mix of diagnoses were quite similar as in reports on FUO [32]. A CRP cut-off point of 10 mg/L resulted in a sensitivity of 100%. In patients with a normal CRP, 18-FDG PET/(CT) was not helpful. For the ESR, when a cut-off point of 20 mm/h was chosen, a sensitivity of 94% and a specificity of 16% were found [32]. 

## 3. Technical Considerations

Related to the broad range of conditions that may cause FUO/IUO it is essential to optimize both the scan procedure and the patient preparation.

- High myocardial uptake of F18-FDG is frequently observed. However as the cause or the focus for FUO or IUO may be localized in or near the heart, this physiologic cardiac uptake may hamper for instance diagnosis of, for example, endocarditis or myocardial sarcoidosis. A fat-allowed, carbohydrate restricted diet starting the day before F18-FDG administration has proven to suppress myocardial F18-FDG uptake satisfactorily [69].
- In patients with FUO/IUO it is important to perform a whole-body PET/CT investigation, including the brain, otherwise a cerebral lymphoma or a rare occult prolactinoma may be missed [70].
- Cold-stimulated F18-FDG uptake by brown adipose tissue (BAT) in humans is more pronounced during fasting. To prevent increased F18-FDG uptake in BAT, which may hamper the interpretation it is advised to have the patient preparation rooms at a comfortable warm temperature [71].
- Start of steroids before F18-FDG PET/(CT) needs to be minimized as much as clinically possible. After corticosteroid administration/immunosuppressive therapy normalisation of F18-FDG uptake in inflammatory lesions is described [72, 73].

### 3.1. Conditions or Situations with Decreased Sensitivity of F-18 FDG Imaging

#### 3.1.1. Hyperglycaemia

Diabetes mellitus (DM) is a common metabolic disorder in the elderly. In diabetic patients, F18-FDG PET/(CT) may be at a disadvantage because peripheral insulin resistance may cause decreased uptake at the site of inflammation [74]. 

The sensitivity of F18-FDG PET/CT in the assessment of malignancy may be reduced by high glucose levels (>180 mg/dL or >10 mmol/L) at the time of the study but not by DM itself. However, no significant impact on the false-negative rate was found in patients with infection and inflammatory processes with either DM or hyperglycaemia [75, 76]. 

#### 3.1.2. Medium- and Small-Vessel Inflammation

Due to the spatial resolution of 4–6 mm F18-FDG PET is less useful in medium- and small-vessel inflammation, in contrast to its utility in LVV. From several reports on the role of F18-FDG-PET in other small- and medium-vessel vasculitis, such as Churg-Strauss syndrome, Wegener's granulomatosis, and polyarteritis nodosa, it may be concluded that these disease entities are detected only when there is large vessel involvement or in case of damage to the adjacent tissues [77–79]. As a consequence of the diameter of the temporal artery F18-FDG PET is in general not suitable for diagnosing isolated temporal arteritis, which may coexist with large vessel involvement of GCA [80].

#### 3.1.3. Prosthetic Joint Infection

Aseptic loosening and infection of a prosthetic joint are both accompanied by an inflammatory response in which leukocytes participate, it is therefore difficult to accurately differentiate with F18-FDG imaging between the 2 conditions [81]. 

In a recent meta-analysis the overall sensitivity and specificity of F18-FDG PET for diagnosing prosthetic joint infection were 82% and 87%, respectively, which is lower than the 90% been reported by numerous investigators, for nearly 30 years, for combined labelled leukocyte/marrow imaging [82, 83]. 

### 3.2. F18-FDG Comparison with Other Techniques

Several reasons make the comparison of the performance of F18-FDG PET/CT and various imaging techniques in FUO and IUO difficult: (1) definition of FUO or IUO may vary among individual patients, the diagnostic workup may differ at different medical facilities, and diagnostic protocols are not standardized worldwide, (2) heterogeneity of the different patient populations, and (3) non-uniformity in PET and CT techniques (including the specific preparation of the patient), and use of contrast material.

Consequently, the percentage of patients for whom no cause can be established using these modalities can range from 10% to 50%. For patients with a negative F18-FDG PET, a limited variety of systemic (non-focal) diseases may still be found through other diagnostic testing, for instance leukaemia. In addition, the calculation of sensitivity and specificity of F18-FDG-PET for patients with FUO or IUO is difficult. 

### 3.3. Socioeconomic Considerations

Despite their increased use of healthcare resources, the management of patients with unexplained symptoms (i.e., FUO an IUO) is perceived as unsatisfactory from the perspective of both the patient and the physician. Also, patients may undergo extensive investigation and medical treatment, which may not only be inappropriate but also hazardous. 

In a retrospective study of 20 patients, costs of the FUO process were determined, including those of the PET/CT investigation, hospitalization days, and complementary tests prior to the PET/CT study. If PET/CT had been performed earlier in the FUO process, assuming the same effectiveness, €5471 per patient would have been saved [84]. 

Hybrid F18-FDG-PET/CT may very well become a cost-effective modality, the high diagnostic yield allows adequate early diagnosis and limits the number of other non-contributing (invasive) tests required and the time to diagnosis, and thereby the duration of hospitalization for diagnostic purposes.

## 4. Conclusions

Both the nonspecificity and the high sensitivity for malignancy, infection, and inflammation of F18-FDG and the inherently superior imaging characteristics of the radioisotope F18 make F18-FDG the ideal radiopharmaceutical for hybrid PET/CT. The synergy of the anatomic-metabolic information offers substantial benefit to patients in the diagnostic workup of FUO and IUO. Although the literature is still scarce, it seems that the diagnostic approaches and the outcomes in patients with FUO can also be applied to patients with IUO. The relatively limited specificity in combination with the high sensitivity for focal diseases make hybrid F18-FDG PET/CT an ideal diagnostic tool to be applied early in the workup to guide the choice for more specific diagnostic examinations. After exclusion of systemic (non-focal) diseases F18-FDG PET/CT has a high negative predictive value and is helpful in identifying patients with benign self-limiting conditions. A negative F18-FDG PET/CT may avoid further diagnostic tests and therapeutic trial with steroids. 

In the uncertainty that remains in which stage of the diagnostic approach to use F18-FDG PET/CT, it has been proven useful in the clinical setting of FUO/IUO. Well-designed future prospective studies are necessary to confirm its efficacy, as F18-FDG PET/CT has the potential to become the routine imaging technique indicating the direction for further diagnostic decisions.

## Figures and Tables

**Table 1:** Helpful contribution of stand-alone F18-FDG PET in FUO.

Author	Study design/technique P/R	Patients number	PPV/NPV^*∧*^	Helpful contribution number/(%)	Final Dx number/(%)
Meller et al. 2004 [53]	Prospective DHC-PET versus Ga-citrate	20 versus 18	92%/75%	11 (**55%**)	18 (**90%**)
Blockmans et al. 2001 [54]	Prospective Full-ring PET versus Ga67-citrate	58 versus 40	*	24 (**41%**)	38 (**66%**)
Lorenzen et al. 2001 [55]	Retrospective Full-ring PET	16	92%/100%	11 (**69%**)	13 (**81%**)
Bleeker-Rovers et al. 2004 [56]	Retrospective Full-ring PET	35	87%/95%	13 (**37%**)	19 (**54**%)
Kjaer et al. 2004 [49]	Prospective Full-ring PET versus In-111 granulocyte	19	30%/67% …	3 (**16%**) (26%)	12 (**63%**)
Buysschaert et al. 2004 [57]	Prospective Full-ring PET	74	36%/*	19 (**26%**)	39 (**53%**)
Bleeker-Rovers et al. 2007 [8]	Prospective multicentre Full-ring PET	70	70%/92%	23 (**33%**)	37 (**51%**)
Jaruskova and Belohlavek 2006 [58]	Retrospective Full-ring PET and PET-CT	124 FUO = 94	*	45 (**36%**)	51 (**84%**)

PET	Total number patients	386		Overall helpfulness of PET 39% (mean)	Overall percentage final diagnosis 67% (mean)

Legends: DHC: dual-headed coincidence camera; NPV: negative predictive value; PPV: positive predictive value; CECT: contrast-enhanced CT; NA: not applicable.

*Data could not be retrieved from the original publication.

^*∧*^NPV is defined as the proportion of patients with negative test results for focal diseases, who are correctly diagnosed.

**Table 2:** Helpful contribution of hybrid F18-FDG PET/CT in FUO.

Author	Study design/technique P/R	Patients number	PPV/NPV	Helpful/contribution number/(%)	Final Dx number/(%)
Federici et al. 2010 [59]	R. Full-ring PET/CT	14	*	7 (**50%**)	10 (**70%**)
Keidar et al. 2008 [60]	P. Full-ring PET/CT	48	81%/100%	22 (**46%**)	28 (**60%**)
Ferda et al. 2010 [61]	R. Full-ring PET/CECT	48	98%/75%^*∧*^	37 (**77%**)	44 (**92%**)
Balink et al. 2009 [62]	R. Full-ring PET/CECT	68	93%/100%	38 (**56%**)	47 (**69%**)
Sheng et al. 2011 [63]	R. Full-ring PET/CECT	48	80%/50%^#^	32 (**67%**)	36 (**75%**)
Pelosi et al. 2011 [64]	R. Full-ring PET/CT	24	85%/91%	11 (**46%**)	17 (**71%**)
Crouzet et al. 2012 [65]	R. Full-ring PET/CT	79	95%/100%	45 (**57%**)	61 (**77%**)

PET/CT	Total number patients	226		Overall helpfulness of PET/CT 57% (mean)	Overall percentage final diagnosis 73% (mean)

Legends: P: prospective; R: retrospective; NPV: negative predictive value; PPV: positive predictive value; CECT: contrast-enhanced CT; NA: not applicable.

*Data could not be retrieved from the original publication.

^*∧*^We question interpretation of results and definition of false negatives. These are based on the later clinical course. However a time window has not been defined for the clinical course. It is therefore possible that the false negatives are explained by another disease process than that was present at the time of PET-CT. This can explain in part the discrepancy between this limited NPV and other publications.”

^#^This low NPV is probably explained by the relatively high prevalence of disease within the study population.
